# Combination of locoregional and systemic therapy for hepatocellular carcinoma with portal vein tumor thrombus: a real-world retrospective study

**DOI:** 10.3389/fonc.2026.1776852

**Published:** 2026-03-10

**Authors:** Xunbo Hou, Linan Yin, RuiBao Liu, Qiannan Xu, Yingchen Li, Bowen Liu, Xuesong Liu

**Affiliations:** 1 Harbin Medical University Cancer Hospital, Harbin, China; 2 Fourth Affiliated Hospital of Harbin Medical University, Harbin, China

**Keywords:** Hepatic arterial infusion chemotherapy, hepatocellular carcinoma, overall survival, PD-1 inhibitors, portal vein tumor thrombosis, progression-free survival, propensity score matching, transarterial chemoembolization

## Abstract

**Background:**

The optimal treatment strategy for advanced hepatocellular carcinoma (HCC) with portal vein tumor thrombosis (PVTT) remains undefined. Although combinations of locoregional therapies—such as transarterial chemoembolization (TACE) and hepatic arterial infusion chemotherapy (HAIC)—with systemic agents (tyrosine kinase inhibitors [TKIs] and PD-1 inhibitors) show promise, direct comparative evidence among different regimens remains limited.

**Methods:**

In this single-center retrospective study, we included 347 patients with unresectable HCC and PVTT treated between January 2020 and December 2022. Patients were categorized into four groups based on initial therapy: TACE-HAIC-TP (n = 79), TACE-TP (n = 90), HAIC-TP (n = 98), and TACE alone (n = 80). The primary endpoints were overall survival (OS) and progression-free survival (PFS).

**Results:**

All combination regimens significantly improved OS and PFS compared with TACE alone (median OS: 11.4 months; median PFS: 5.8 months; all p < 0.001). The TACE-HAIC-TP group had the longest median OS (21.0 months) and PFS (15.3 months). However, after propensity score matching, no significant difference in survival outcomes was observed between the TACE-HAIC-TP and HAIC-TP groups. The HAIC-TP and TACE-TP regimens demonstrated comparable efficacy. Regarding safety, TACE-HAIC-TP was associated with the highest incidence of adverse events, including appetite loss, fatigue, nausea/vomiting, bleeding, and immune-related pneumonia. HAIC-TP carried a higher risk of gastrointestinal reactions and bleeding, whereas hand-foot syndrome was more frequent with TACE-TP.

**Conclusion:**

In patients with unresectable HCC and PVTT, combining TKIs and PD-1 inhibitors with locoregional therapy (TACE or HAIC) confers superior survival benefits over TACE monotherapy. The HAIC-TP regimen was associated with a more favorable balance of efficacy and tolerability compared with the more intensive TACE-HAIC-TP strategy, suggesting it may represent a promising therapeutic option pending prospective validation. Treatment selection should be individualized based on efficacy–safety trade-offs.

## Introduction

Hepatocellular carcinoma (HCC) is a leading cause of cancer-related mortality worldwide (1). In 10%–40% of patients, HCC presents with portal vein tumor thrombus (PVTT)—a marker of aggressive biology associated with rapid hepatic deterioration, exacerbated portal hypertension, and a median overall survival of only 3 months (2–6). The management of PVTT demands integrated strategies that simultaneously address local tumor control, systemic dissemination, and the immunosuppressive microenvironment—a paradigm increasingly adopted across oncology (7).

Locoregional therapies such as transarterial chemoembolization (TACE) and hepatic arterial infusion chemotherapy (HAIC) provide cytoreduction and may modulate antitumor immunity through immunogenic cell death or sustained antigen release. When combined with systemic “targeted–immunotherapy”—defined here as concurrent tyrosine kinase inhibitors (TKIs) and PD-1 inhibitors (hereafter “TP”)—these approaches aim to synergize local control, vascular normalization, and T-cell reinvigoration (3, 8–11).While HAIC with FOLFOX has shown superiority over TACE alone in PVTT (12, 13), and sequential TACE-HAIC may enhance intrahepatic and thrombus control (14, 15), the optimal regimen intensity remains unclear. Direct comparisons between the triplet combination (HAIC-TP) and the more intensive quadruplet approach (TACE-HAIC-TP) are lacking, particularly regarding the balance of efficacy, safety, and procedural feasibility in this high-risk population.

This retrospective cohort study aimed to address this gap by systematically comparing the efficacy and safety of four treatment regimens—TACE-HAIC-TP, TACE-TP, HAIC-TP, and TACE monotherapy—in 347 patients with unresectable HCC and PVTT (Vp1–Vp4).These findings support HAIC-TP as a favorable strategy that balances efficacy and tolerability, providing clinically relevant evidence to guide individualized treatment decisions for this difficult-to-treat population.

## Materials and methods

### Study design and patient population

This single-center, retrospective cohort study was approved by the Ethics Committee of the Cancer Hospital of Harbin Medical University (Approval No.: YD2024-11). Informed consent was waived owing to the retrospective design. The study adhered to the ethical principles of the Declaration of Helsinki.The study protocol was registered retrospectively in the Chinese Clinical Trial Registry (ChiCTR2400091091).

We retrospectively identified 473 patients with primary hepatocellular carcinoma (HCC) and concomitant portal vein tumor thrombus (PVTT) who received initial treatment at the Department of Interventional Therapy between January 2020 and December 2022.Following further review for data completeness and treatment regularity, a final cohort of 347 patients​ was included in the analysis.HCC diagnosis was established according to the clinical or pathological criteria of the Chinese National Liver Cancer (CNLC) Guidelines (3).

Inclusion criteria were: (i) age ≥18 years; (ii) HCC confirmed by clinical history, imaging characteristics, and/or histopathology; (iii) at least one measurable intrahepatic lesion per mRECIST v1.1; (iv) Barcelona Clinic Liver Cancer (BCLC) stage C; (v) Child–Pugh class A or B liver function; and (vi) Eastern Cooperative Oncology Group Performance Status (ECOG PS) score ≤2.

Exclusion criteria comprised: (i) prior surgical resection or local ablative therapy; (ii) decompensated cirrhosis (Child–Pugh class C, refractory ascites, or overt hepatic encephalopathy); (iii) incomplete laboratory or imaging data; (iv) concurrent primary malignancy; (v) irregular treatment or loss to follow-up; and (vi) severe cardiovascular, hematological, or renal comorbidities.

After applying exclusion criteria, 347 patients were included and stratified into four groups based on initial treatment regimen:

Group 1 (TACE–HAIC–TP, n=79): Transarterial chemoembolization (TACE) combined with hepatic arterial infusion chemotherapy (HAIC), tyrosine kinase inhibitors (TKIs), and PD-1 inhibitors;

Group 2 (TACE–TP, n=90): TACE plus TKIs and PD-1 inhibitors;

Group 3 (HAIC–TP, n=98): HAIC plus TKIs and PD-1 inhibitors;

Group 4 (TACE, n=80): TACE monotherapy.

**Figure 1:**
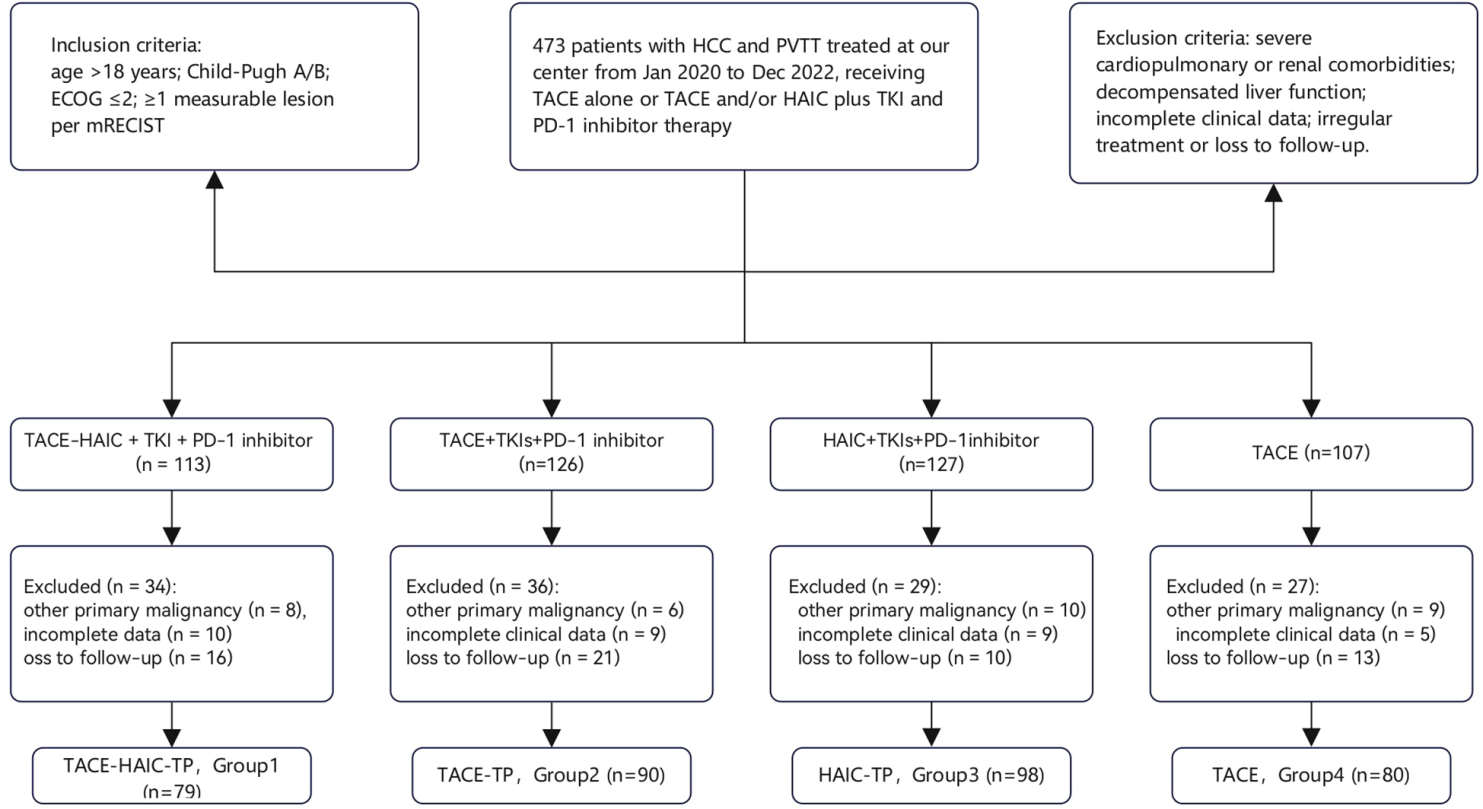
Case collection flow chart.

Here, “TP” denotes combined systemic therapy with TKIs (lenvatinib or donafenib) and PD-1 inhibitors (camrelizumab or sintilimab). The patient selection workflow is illustrated in **Figure 1**.

### Treatment protocols

#### TACE and HAIC

All vascular interventions were performed via femoral artery access. Following diagnostic angiography of the celiac trunk and hepatic arteries, superselective catheterization of tumor-feeding vessels was achieved using a 2.5F microcatheter.

For TACE, an emulsion of iodized oil (5–20 mL) mixed with epirubicin or pirarubicin (30 mg/m²) and oxaliplatin or lobaplatin (50 mg) was administered. In cases of hypervascular or large solitary tumors, partial embolization was performed to achieve incomplete devascularization, followed by catheter retention for subsequent HAIC.

The TACE–HAIC combination protocol involved initial chemoembolization as above, followed by continuous HAIC via an indwelling catheter using a modified FOLFOX regimen: oxaliplatin (85 mg/m² over 2 h), leucovorin (400 mg/m² over 2 h), bolus 5-fluorouracil (5-FU, 400 mg/m²), and continuous 5-FU infusion (2400 mg/m² over 46 h or 1200 mg/m² over 23 h). Treatment cycles were repeated every 3–4 weeks.

Prior to each cycle, patients underwent comprehensive assessment including contrast-enhanced liver CT or MRI, chest CT, electrocardiography, and laboratory tests (liver and renal function, serum electrolytes, glucose, myocardial enzymes, complete blood count, coagulation profile, thyroid function, AFP, CEA, and CA19-9). Chemotherapy doses were individually adjusted based on treatment history, laboratory parameters, and clinical status.

#### Systemic therapy

TKIs consisted of lenvatinib (12 mg/day for body weight ≥60 kg; 8 mg/day if <60 kg) or donafenib (200 mg twice daily). PD-1 inhibitors included camrelizumab or sintilimab (200 mg intravenously). Systemic therapy was initiated on post-procedural day 1 following the assigned locoregional intervention and continued until radiological progression or unacceptable toxicity.

### Treatment response and safety assessment

Tumor response was evaluated 3–4 weeks post-treatment initiation by contrast-enhanced CT or MRI, interpreted independently by two blinded radiologists according to mRECIST v1.1. Discrepancies were resolved by consensus or adjudication by a third blinded reviewer.

Adverse events (AEs) were recorded throughout the treatment course, graded according to the Common Terminology Criteria for Adverse Events (CTCAE) version 5.0, and reported as the highest grade observed per patient.

### Follow-up

Patients were monitored monthly during the first year and subsequently every 3–4 months. Each visit included imaging (CT/MRI), serum AFP, liver function tests, complete blood count, and coagulation assays.

### Statistical analysis

Analyses were performed using Python (v3.10) and R (v4.5.2). Continuous variables were expressed as mean ± SD or median (interquartile range) and compared using Student’s t-test or Mann–Whitney U test. Categorical variables were presented as frequencies (%) and analyzed by χ² or Fisher’s exact test. Survival curves were generated via Kaplan–Meier method and compared using the log-rank test. Multivariate analysis employed Cox proportional hazards regression. To address potential selection bias between Group 1 and Group 3, 1:1 propensity score matching was conducted using nearest-neighbor matching with a caliper of 0.2, adjusting for age, sex, Child–Pugh class, ECOG PS, tumor size, tumor number, and baseline AFP. All tests were two-sided; p< 0.05 was considered statistically significant.

### Baseline characteristics

A total of 347 patients were included in the final analysis and categorized into four groups based on their initial treatment regimens. The baseline demographic and clinical characteristics of the four groups are summarized in **Table 1**. No statistically significant differences were observed​ among the groups in terms of age, sex distribution, liver function parameters (Child-Pugh class, TB, AST, ALT, PT, albumin), tumor burden (number of tumors, maximum tumor diameter, PVTT Vp grade, extrahepatic metastasis), or AFP levels (all p>0.05). Although numerical variations were noted in hepatitis background (particularly HCV infection rate) and ECOG PS score, these differences did not reach statistical significance. These findings indicate that the baseline characteristics were well-balanced across the four groups, providing a reliable foundation for subsequent intergroup comparisons of efficacy and safety outcomes.

**Table 1:** Baseline characteristics of major clinical parameters in four patient groups.

Characteristic	Group1 (n=79)	Group2 (n=90)	Group3 (n=98)	Group4 (n=80)	p
Sex, n (%)					
Male	71 (89.8)	77 (85.5)	84 (85.7)	65 (81.2)	0.642
Female	8 (10.1)	13 (14.4)	14 (14.3)	15 (18.8)	
Age					
x̄; ± s (years)	55.1 ± 9.87	55.2 ± 10.5	58.1 ± 8.6	56.3 ± 8.8	0.127
> 55 years, n (%)	40 (50.6)	40 (44.4)	54 (55.1)	40 (50)	0.507
Liver function, Child-Pugh, n (%)					
A	61 (77.2)	77 (85.6)	77 (78.5)	65 (81.2)	0.493
B	18 (22.8)	13 (14.4)	21 (21.4)	15 (18.8)	
ECOG PS, n (%)					
0-1	50 (63.2)	46 (51.2)	50 (51.0)	41 (51.2)	0.261
2	29 (36.7)	44 (48.8)	48 (48.9)	39 (48.8)	
Hepatitis, n (%)					
Yes	74 (93.6)	75 (83.3)	84 (85.7)	70 (87.5)	0.106
No	5 (6.3)	15 (16.7)	14 (14.3)	10 (12.5)	
HBV (+)	62 (78.4)	71 (78.8)	70 (71.4)	61 (76.2)	0.695
HCV (+)	12 (15.1)	6 (6.7)	15 (15.3)	9 (11.2)	0.108
Liver cirrhosis, n (%)					
Yes	59 (74.7)	67 (74.4)	73 (74.4)	61 (76.2)	0.991
No	20 (25.3)	23 (25.6)	25 (25.6)	19 (23.8)	
Number of tumors, n (%)					
< 3	33 (41.8)	47 (52.2)	53 (54.0)	39 (48.7)	0.362
≥ 3	46 (58.2)	43 (47.7)	45 (46.0)	41 (51.2)	
Maximum tumor diameter, n (%)					
< 5 cm	19 (24.1)	21 (23.3)	15 (15.3)	15 (18.8)	0.308
≥ 5 cm	60 (75.9)	69 (76.6)	83 (84.6)	65 (81.2)	
PVTT Vp grade, n (%)					
Vp2,3	50 (63.3)	48 (53.3)	63 (64.3)	50 (62.5)	0.366
Vp4	29 (36.7)	42 (46.6)	35 (35.7)	30 (37.5)	
Extrahepatic metastasis, n (%)					
Yes	15 (18.9)	19 (21.1)	15 (15.3)	16 (20.0)	0.759
No	64 (81.0)	71 (78.9)	83 (84.6)	64 (80.0)	
AFP, n (%)					
≤ 400 ng/mL	32 (40.5)	46 (51.1)	53 (54.1)	43 (53.8)	0.108
> 400 ng/mL	47 (59.4)	44 (48.8)	45 (45.9)	37 (46.2)	
Laboratory examination indicators, x̄; ± s					
TB, (μmol/L)	21.2 ± 8.2	22.7 ± 11.1	23.2 ± 13.1	23.6 ± 8.5	0.451
AST, (U/L)	65.9 ± 30.0	62.8 ± 37.6	70.5 ± 53.4	67.4 ± 35.3	0.622
ALT, (U/L)	51.3 ± 22.2	49.8 ± 39.1	51.8 ± 42.8	50.8 ± 34.8	0.781
PT, (s)	12.8 ± 1.6	12.5 ± 1.0	12.5 ± 1.1	12.6 ± 1.2	0.533
ALB, (g/L)	37.6 ± 3.5	39.0 ± 4.9	37.8 ± 4.9	39.1 ± 3.5	0.087

### Treatment-related adverse events

The safety profiles of the four treatment regimens are summarized in **Table 2**. Overall, the spectrum and incidence of treatment-related adverse events (AEs) varied significantly among the groups, reflecting the intensity of the respective treatment strategies.

**Table 2:** Summary of treatment-related adverse events across four groups.

AE Type n(%)	Group1 (n=79)	Group2 (n=90)	Group3 (n=98)	Group4 (n=80)
Weight loss	31 (39.2)	37 (41.1)	46 (46.9)	32 (40.0)
Decreased appetite	43 (54.4)	28 (31.1)	40 (40.8)	28 (35.0)
Fatigue	50 (63.3)	41 (45.6)	45 (45.9)	29 (36.2)
Abdominal pain	43 (54.4)	21 (23.3)	40 (40.8)	34 (42.5)
Diarrhea	35 (44.3)	32 (35.6)	36 (36.7)	25 (31.2)
Nausea and vomiting	36 (45.5)	17 (18.9)	35 (35.7)	22 (27.5)
Ascites	28 (35.4)	26 (28.9)	33 (33.7)	6 (7.5)
Rash	30 (37.9)	28 (31.1)	25 (25.5)	3 (3.7)
Hand-foot syndrome	19 (24.1)	25 (27.8)	14 (14.3)	0 (0.0)
Oral mucositis	22 (27.8)	13 (14.4)	12 (12.2)	9 (11.2)
Arthralgia	14 (17.7)	8 (8.9)	9 (9.2)	7 (8.7)
Bleeding event	20 (25.3)	4 (4.4)	28 (28.6)	4 (5.0)
Immune-related pneumonitis	9 (11.4)	1 (1.1)	9 (9.2)	0 (0.0)

The incidence of AEs was generally higher in the combination therapy groups (Groups 1-3) compared to the TACE-alone group (Group 4). The most intensive regimen, TACE-HAIC-TP (Group 1), demonstrated the highest incidence rates for multiple AEs, including fatigue (63.3%), decreased appetite (54.4%), abdominal pain (54.4%), nausea/vomiting (45.5%), and rash (37.9%). The HAIC-TP regimen (Group 3) was associated with a higher risk of bleeding events (28.6%). Notably, the TACE-TP regimen (Group 2) exhibited a relatively distinct safety profile, with the lowest incidence of immune-related pneumonia (1.1%) and bleeding events (4.4%) among the triple regimens, but a higher rate of hand-foot syndrome (27.8%).

Most AEs were grade 1–2 and clinically manageable. However, grade 3 or higher AEs, such as bleeding and immune-related pneumonia, occurred more frequently in the combination therapy groups, particularly in Groups 1 and 3.

In summary, increasing treatment intensity was accompanied by elevated safety risks. The HAIC-TP and TACE-TP regimens demonstrated distinct safety profiles, which should be carefully considered in clinical decision-making alongside efficacy data.

## Results

### Comparison of all groups on survival outcomes

**Figure 2:**
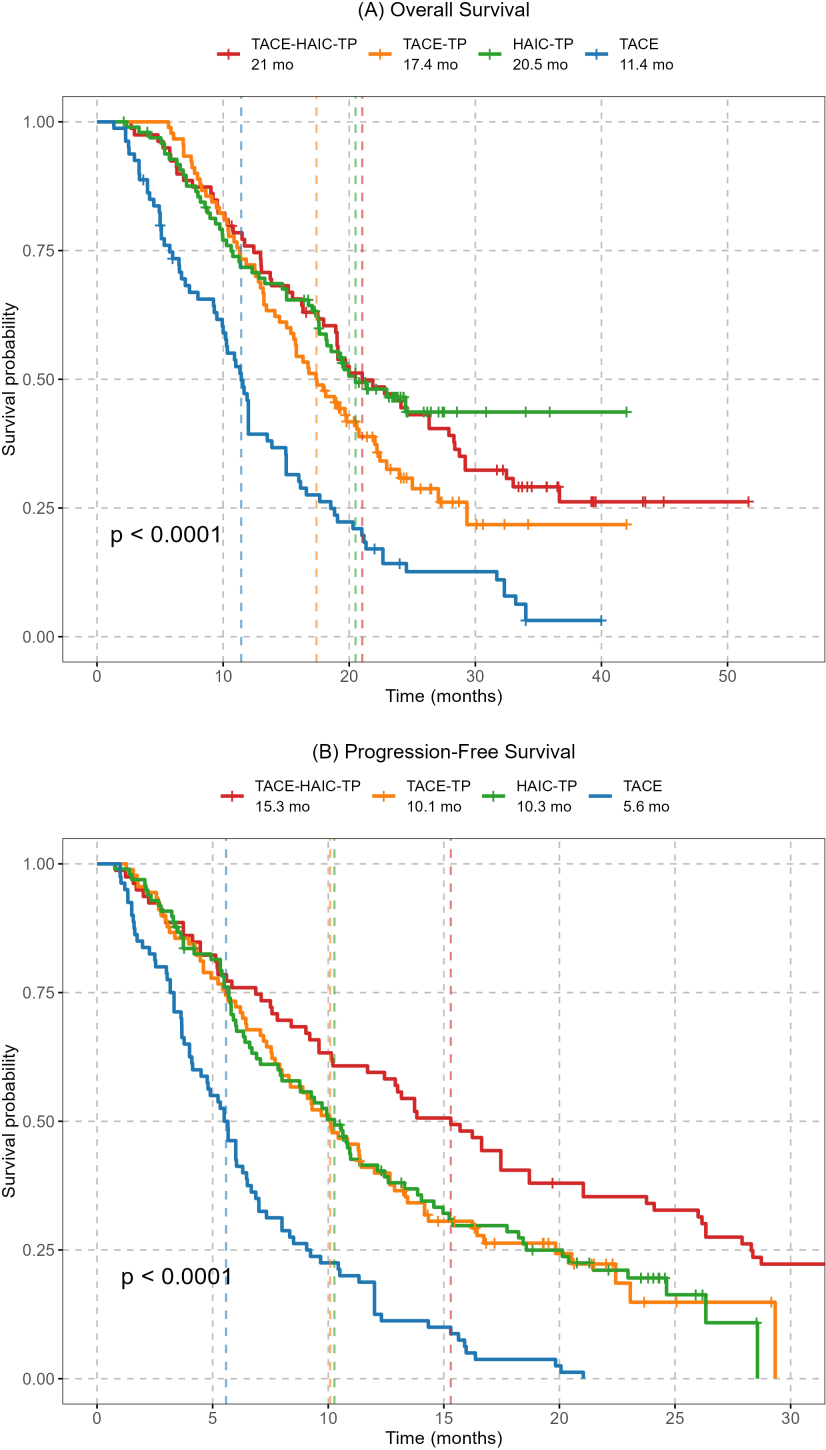
Overall survival (OS) and progression-free survival (PFS) by treatment regimen in patients with advanced hepatocellular carcinoma and portal vein tumor thrombosis. **(A)** The overall survival curve shows that the TACE-HAIC-TP group had a median OS of 21.0 months, significantly longer than TACE-TP (17.4 months), HAIC-TP (20.5 months), and TACE alone (11.4 months); **(B)** The progression-free survival curve indicates a median PFS of 15.3 months for the TACE-HAIC-TP group, which was also superior to the other regimens. All combination therapies demonstrated statistically significant improvements in both OS and PFS compared to TACE monotherapy (log-rank test, p< 0.0001), with the TACE-HAIC-TP cohort exhibiting the most favorable outcomes. Shaded areas represent 95% confidence intervals; vertical dashed lines denote median survival times.

The Kaplan-Meier curves for overall survival (OS) and progression-free survival (PFS) across all treatment groups are presented in **Figure 2**. The survival analysis revealed that all combination regimens significantly improved both OS and PFS compared with TACE monotherapy (median OS: 11.4 months; median PFS: 5.8 months; all p<0.001, log-rank test). Among the combination therapies, the TACE-HAIC-TP group (Group 1) demonstrated the longest median OS (21.0 months) and PFS (15.3 months) in the overall cohort analysis.

To quantify the treatment effects, Cox proportional hazards regression analyses were performed, with the TACE monotherapy group (Group 4) as the reference (**Table 3**). The results confirmed significant reductions in the risk of death and disease progression for all combination therapies. The hazard ratios (HR) for OS were as follows: TACE-TP group (HR = 0.51, 95% CI 0.36-0.72, p< 0.001), HAIC-TP group (HR = 0.37, 95% CI 0.26-0.53, p< 0.001), and TACE-HAIC-TP group (HR = 0.39, 95% CI 0.28-0.56, p< 0.001). Similarly, the HRs for PFS were significantly reduced across all combination groups. These results robustly demonstrate the superior efficacy of combining locoregional therapy (TACE or HAIC) with systemic therapy (TKIs and PD-1 inhibitors) over TACE monotherapy in this patient population with advanced HCC and PVTT.

**Table 3:** Association of treatment groups with overall survival and progression-free survival (reference: TACE).

Treatment group	Overall survival (OS)	p	Progression-free survival (PFS)	p
	HR (95% CI)		HR (95% CI)	
TACE-TP	0.51 (0.36–0.72)	<0.001	0.42 (0.31–0.59)	<0.001
HAIC-TP	0.37 (0.26–0.53)	<0.001	0.42 (0.30–0.58)	<0.001
TACE-HAIC-TP	0.39 (0.28–0.56)	<0.001	0.27 (0.19–0.38)	<0.001

HR denotes hazard ratio, 95% CI denotes 95% confidence interval; reference group is the TACE group; all p<0.001.

### Head-to-head comparisons between key combination regimens

#### Comparative efficacy and safety of TACE-HAIC-TP versus HAIC-TP

**Figure 3:**
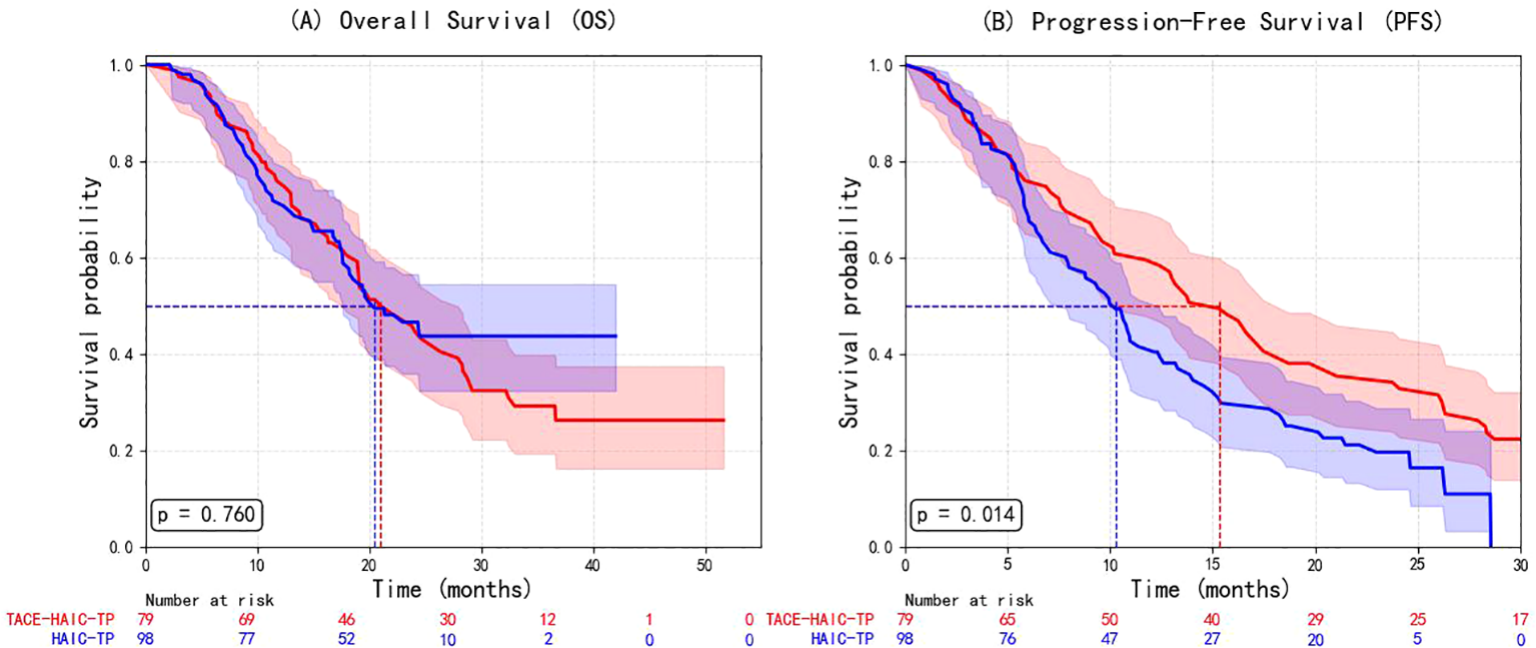
Kaplan–Meier survival curves comparing **(A)** overall survival (OS) and **(B)** progression-free survival (PFS) between patients treated with TACE plus HAIC, tyrosine kinase inhibitors (TKIs), and PD-1 inhibitors (group 1) and those treated with HAIC plus TKIs and PD-1 inhibitors alone (group 3). The log-rank test showed no significant difference in OS (p= 0.760), but a statistically significant improvement in PFS (p= 0.014) in the TACE-HAIC-TP group. TACE, transarterial chemoembolization; HAIC, hepatic arterial infusion chemotherapy; OS, overall survival; PFS, progression-free survival.

In the original cohort, patients receiving TACE-HAIC-TP exhibited significantly longer progression-free survival (PFS) compared to those treated with HAIC-TP (median PFS: 15.3 vs. 10.3 months; log-rank p= 0.014), whereas overall survival (OS) was comparable between groups (p= 0.760) (**Figures 3A, B**).

**Figure 4:**
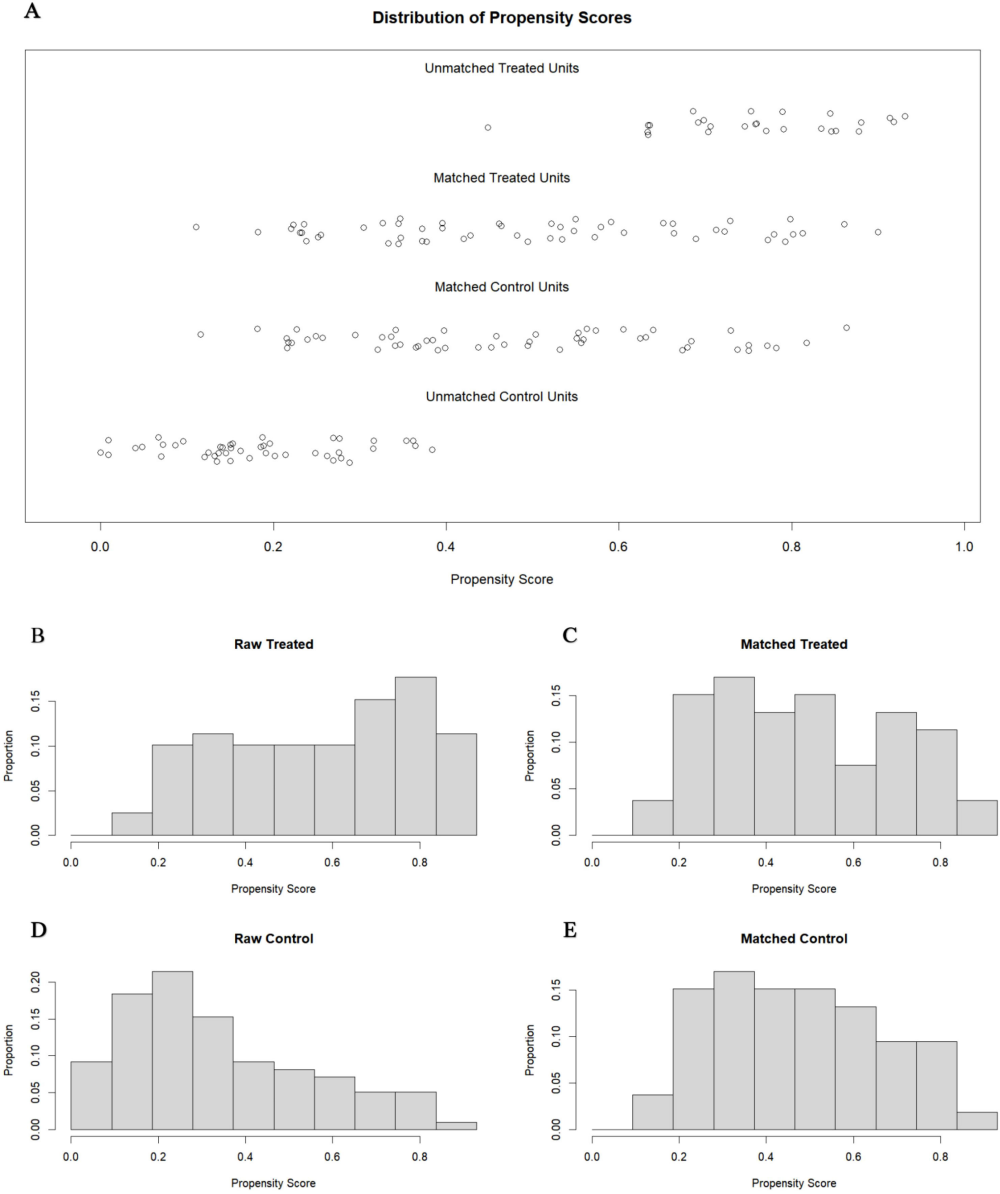
Distribution of samples and propensity scores before and after Matching. **(A)** Scatter plot of sample distribution before and after matching. **(B–E)** Histograms of propensity score distribution before and after matching.

To improve comparability between groups, we performed 1:1 propensity score matching, resulting in 53 well-balanced pairs (standardized mean differences <0.1 for all covariates) (**Table 4**, **Figure 4**).

**Table 4:** Comparison of baseline characteristics between TACE-HAIC-TP and HAIC-TP groups before and after propensity score matching.

Variables	Before matching		p	After matching		p	SMD
	TACE-HAIC-TP (n=79)	HAIC-TP (n=98)		TACE-HAIC-TP (n=53)	HAIC-TP (n=53)		
Age, years	55.1 ± 9.87	58.1 ± 8.6	0.127	57.00 ± 10.05	56.11 ± 8.27	0.621	0.096
Age >55	40 (50.6)	54 (55.1)	0.215	31 (58.5%)	28 (52.8%)	0.696	0.094
Male	71 (89.8)	84 (85.7)	0.089	45 (84.9%)	46 (86.8%)	1.000	0.054
ECOG ≥2	29 (36.7)	48 (48.9)	0.342	23 (43.4%)	21 (39.6%)	0.844	0.077
HBV+	62 (78.4)	70 (71.4)	0.156	41 (77.4%)	40 (75.5%)	1.000	0.044
HCV+	12 (15.1)	15 (15.3)	0.451	8 (15.1%)	9 (17.0%)	1.000	0.051
Hepatitis	74 (93.6)	84 (85.7)	0.267	49 (92.5%)	49 (92.5%)	1.000	<0.001
Cirrhosis	59 (74.7)	73 (74.4)	0.198	40 (75.5%)	42 (79.2%)	0.816	0.090
Tumors ≥3	46 (58.2)	45 (46.0)	0.073	28 (52.8%)	30 (56.6%)	0.845	0.076
Size ≥5 cm	60 (75.9)	83 (84.6)	0.394	42 (79.2%)	41 (77.4%)	1.000	0.046
PVTT Vp4	29 (36.7)	35 (35.7)	0.521	20 (37.7%)	16 (30.2%)	0.538	0.160
Metastasis	15 (18.9)	15 (15.3)	0.178	9 (17.0%)	8 (15.1%)	1.000	0.051
AFP >400	47 (59.4)	45 (45.9)	0.289	25 (47.2%)	23 (43.4%)	0.845	0.076
Child-Pugh B	18 (22.8)	21 (21.4)	0.436	12 (22.6%)	14 (26.4%)	0.821	0.088
TB, μmol/L	21.2 ± 8.2	23.2 ± 13.1	0.063	20.7 ± 8.5	20.8 ± 9.0	0.930	0.017
AST, U/L	65.9 [35.4, 96.4]	70.5 [17.1, 123.9]	0.182	67.4 [46.5, 89.7]	61.0 [42.0, 88.0]	0.473	0.003
ALT, U/L	51.3 [29.1, 73.5]	51.8 [9.0, 94.6]	0.224	60.3 [42.5, 73.7]	40.0 [25.0, 60.0]	0.053	0.078
PT, s	12.8 ± 1.6	12.5 ± 1.1	0.157	12.59 ± 1.47	12.57 ± 1.07	0.910	0.022
Albumin, g/L	37.6 ± 3.5	37.8 ± 4.9	0.095	34.62 ± 3.63	34.97 ± 4.56	0.667	0.084

**Figure 5:**
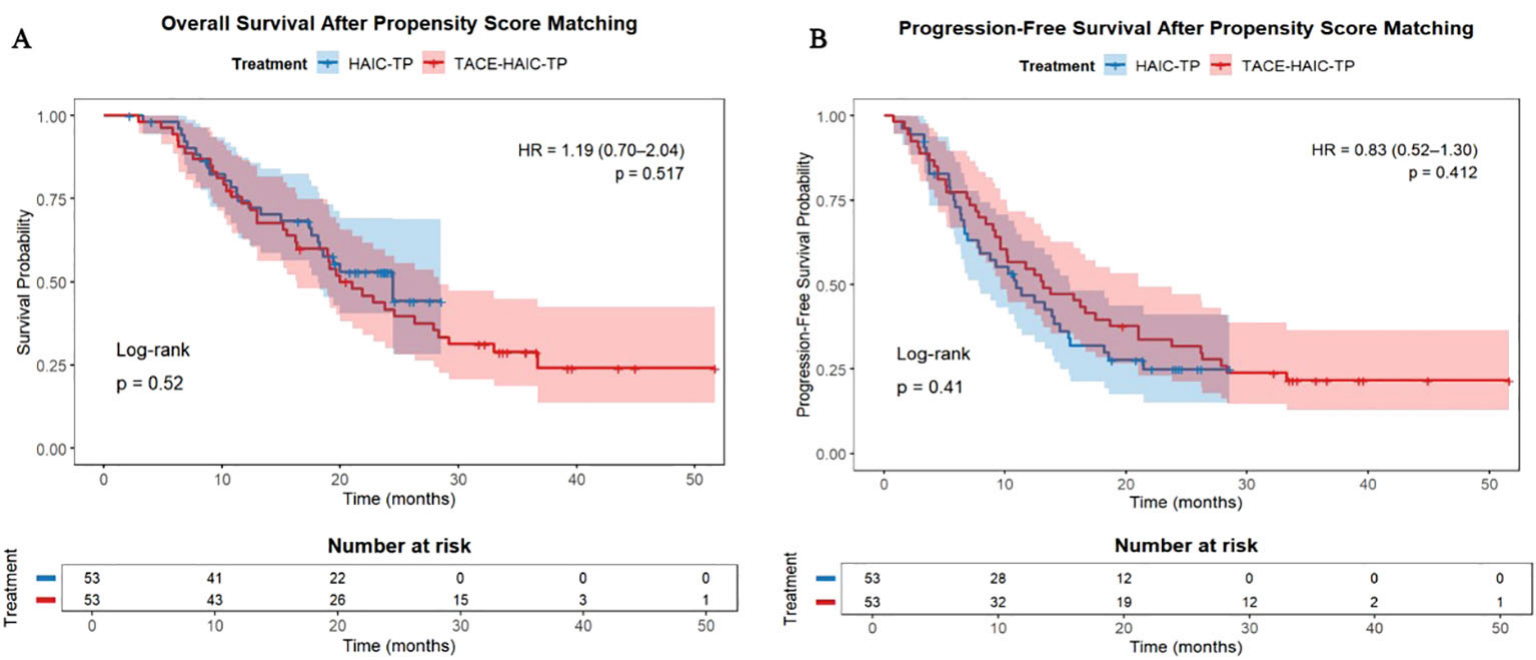
Kaplan-Meier survival curves after propensity score matching. **(A)** Overall survival (OS); **(B)** Progression-free survival (PFS).

After matching, the PFS advantage of TACE-HAIC-TP was no longer statistically significant (HR = 0.83, 95% CI: 0.52–1.30; p=0.410), and OS remained similar (p> 0.05) (**Figures 5A, B**). These findings suggest that the apparent early benefit in PFS observed in the raw analysis was likely attributable to confounding by indication or baseline heterogeneity.

With respect to safety, TACE-HAIC-TP was associated with significantly higher rates of oral mucositis (27.8% vs. 12.2%; p= 0.011) and fatigue (63.3% vs. 45.9%; p= 0.031). Given the lack of survival benefit after adjustment and an increased toxicity burden, HAIC-TP represents a more favorable therapeutic option in terms of risk–benefit balance for most patients with unresectable HCC and PVTT (**Table 5**).

**Table 5:** Treatment-related adverse events in the TACE-HAIC combined with TKIs and PD-1 inhibitor group (group 1) versus the HAIC combined with TKIs and PD-1 inhibitor group (Group 3).

AE type n (%)	Group1 (n=79)	Group3 (n=98)	p
Weight loss	31 (39.2)	46 (46.9)	0.368
Decreased appetite	43 (54.4)	40 (40.8)	0.097
Fatigue	50 (63.3)	45 (45.9)	0.031
Abdominal pain	43 (54.4)	40 (40.8)	0.098
Diarrhea	35 (44.3)	36 (36.7)	0.385
Nausea and vomiting	36 (45.5)	35 (35.7)	0.239
Ascites	28 (35.4)	33 (33.7)	0.887
Rash	30 (37.9)	25 (25.5)	0.105
Hand-foot syndrome	19 (24.1)	14 (14.3)	0.143
Oral mucositis	22 (27.8)	12 (12.2)	0.011
Arthralgia	14 (17.7)	9 (9.2)	0.127
Bleeding event	20 (25.3)	28 (28.6)	0.732
Immune-related pneumonitis	9 (11.4)	9 (9.2)	0.749

### TACE-TP versus HAIC-TP: equivalent efficacy, divergent toxicity profiles

**Figure 6:**
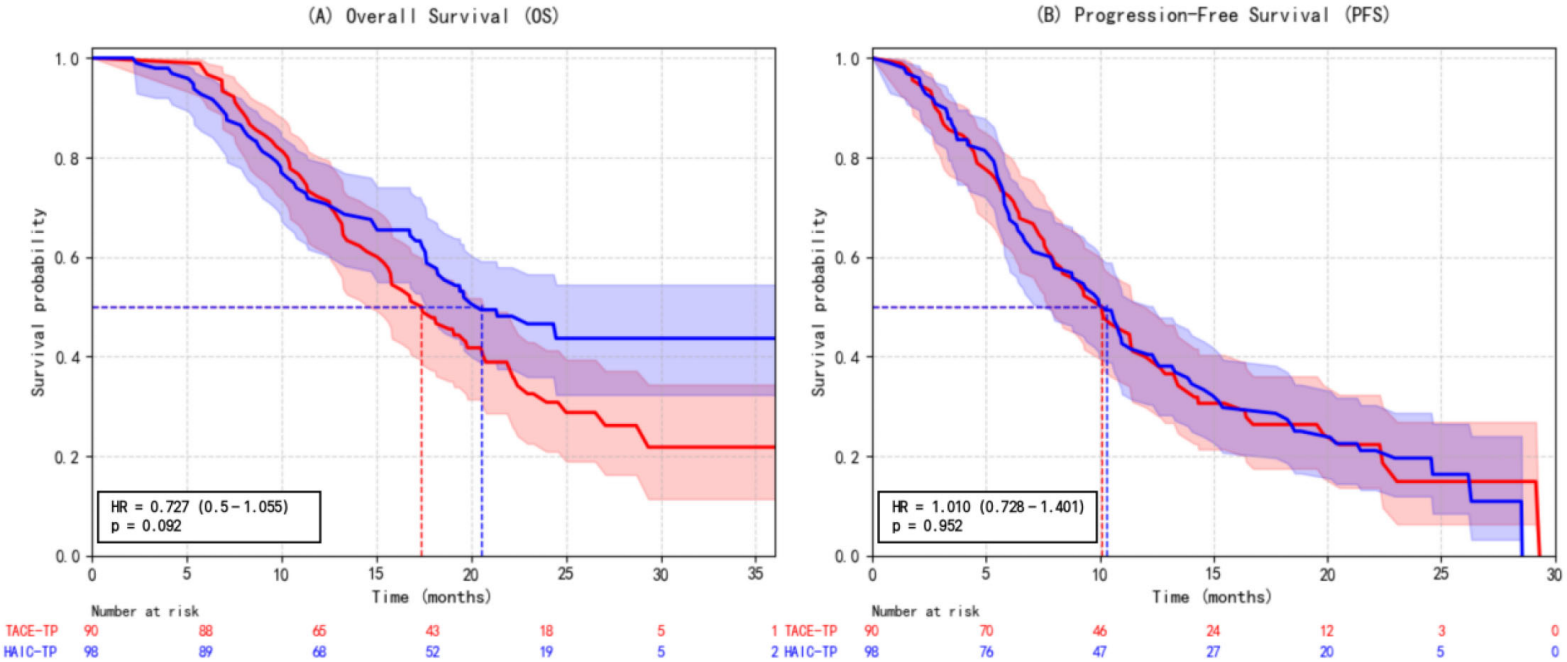
Kaplan-Meier survival curves comparing **(A)** overall survival (OS) and **(B)** progression-free survival (PFS) between patients treated with TACE plus tyrosine kinase inhibitors (TKIs) and PD-1 inhibitors (group 2) and those treated with HAIC plus TKIs and PD-1 inhibitors (group 3). Hazard ratio (HAIC vs. TACE): 0.727; 95% CI: 0.500–1.055; p= 0.092 for OS; and hazard ratio (HAIC vs. TACE): 1.010; 95% CI: 0.728–1.401; p= 0.952 for PFS. No statistically significant differences were observed in either OS or PFS between the two groups (log-rank test). TACE, transarterial chemoembolization; HAIC, hepatic arterial infusion chemotherapy; OS, overall survival; PFS, progression-free survival; HR, hazard ratio; CI, confidence interval.

TACE-TP and HAIC-TP demonstrated comparable survival outcomes. Specifically, HAIC-TP was associated with a non-significant trend toward improved overall survival compared to TACE-TP (HR = 0.727, 95% CI: 0.500–1.055; p= 0.092), with median OS of 20.5 months versus 17.4 months, respectively. Progression-free survival was nearly identical between the two regimens (HR = 1.010, 95% CI: 0.728–1.401; p= 0.952; median PFS: 10.3 vs. 10.1 months) (**Figure 6**).

However, their safety profiles differed markedly: TACE-TP was more frequently associated with hand-foot syndrome (27.8% vs. 14.3%; p= 0.020), whereas HAIC-TP carried higher risks of gastrointestinal toxicity (nausea/vomiting: 35.7% vs. 18.9%; p= 0.009), bleeding (28.6% vs. 4.4%; p< 0.001), and immune-related pneumonia (9.2% vs. 1.1%; p= 0.012). These findings support regimen selection based on individual comorbidities and tolerance rather than efficacy alone (**Table 6**).

**Table 6:** Treatment-related adverse events in the TACE combined with TKIs and PD-1 inhibitor group (group 2) versus the HAIC combined with TKIs and PD-1 inhibitor group (group 3).

AE type n(%)	Group2 (n=90)	Group3 (n=98)	p
Weight loss	37 (41.1)	46 (46.9)	0.420
Decreased appetite	28 (31.1)	40 (40.8)	0.159
Fatigue	41 (45.6)	45 (45.9)	0.962
Abdominal pain	21 (23.3)	40 (40.8)	0.010
Diarrhea	32 (35.6)	36 (36.7)	0.864
Nausea and vomiting	17 (18.9)	35 (35.7)	0.009
Ascites	26 (28.9)	33 (33.7)	0.477
Rash	30 (33.3)	25 (25.5)	0.235
Hand-foot syndrome	25 (27.8)	14 (14.3)	0.020
Oral mucositis	13 (14.4)	12 (12.2)	0.669
Arthralgia	8 (8.9)	9 (9.2)	0.950
Bleeding event	4 (4.4)	28 (28.6)	<0.001
Immune-related pneumonitis	1 (1.1)	9 (9.2)	0.012

#### Impact of adding HAIC to TACE-TP

**Figure 7:**
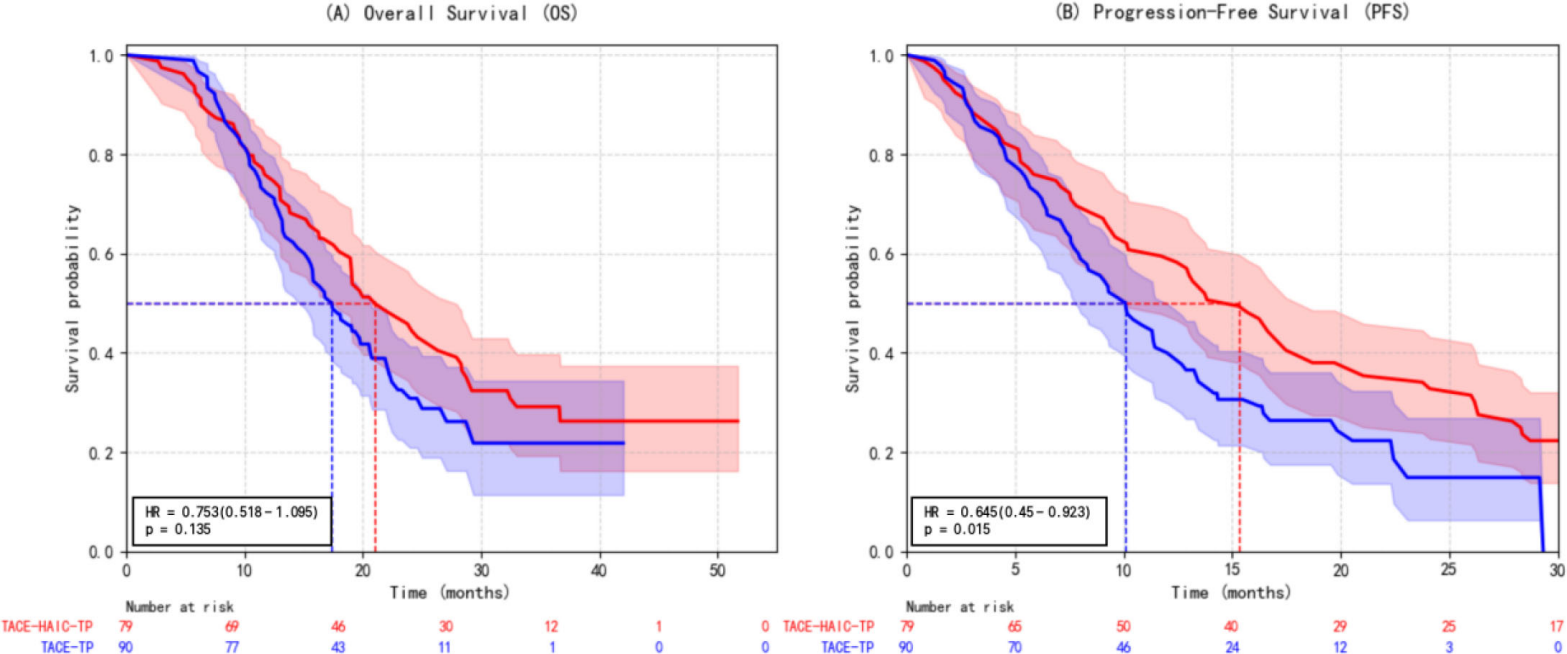
Kaplan-Meier survival curves comparing **(A)** overall survival (OS) and **(B)** progression-free survival (PFS) between patients treated with TACE plus HAIC, tyrosine kinase inhibitors (TKIs), and PD-1 inhibitors (group 1) and those treated with TACE plus TKIs and PD-1 inhibitors alone (group 2). Hazard ratio (TACE+HAIC vs. TACE): 0.753; 95% CI: 0.518–1.095; p=0.135 for OS; and hazard ratio (TACE+HAIC vs. TACE): 0.645; 95% CI: 0.450–0.923; p=0.015 for PFS. The log-rank test showed no significant difference in OS, but a statistically significant improvement in PFS in the TACE-HAIC-TP group. TACE, transarterial chemoembolization; HAIC, hepatic arterial infusion chemotherapy; OS, overall survival; PFS, progression-free survival; HR, hazard ratio; CI, confidence interval.

The addition of HAIC to TACE-TP (i.e., TACE-HAIC-TP) did not confer an overall survival benefit compared to TACE-TP alone (HR = 0.753, 95% CI: 0.518–1.095; p=0.135). However, it significantly prolonged progression-free survival (HR = 0.645, 95% CI: 0.450–0.923; p=0.015), suggesting enhanced short-term tumor control (**Figure 7**).

This PFS advantage came at the cost of markedly increased toxicity. Patients receiving TACE-HAIC-TP experienced significantly higher rates of appetite loss (54.4% vs. 31.1%; p=0.002), abdominal pain (54.4% vs. 23.3%; p<0.001), nausea/vomiting (45.5% vs.18.9%; p<0.001), bleeding (25.3% vs. 4.4%; p<0.001), and immune-related pneumonia (11.4% vs.1.1%; p=0.007). Given this unfavorable risk–benefit trade-off in the absence of an OS gain, TACE-HAIC-TP should be reserved for highly selected patients with high tumor burden who are capable of tolerating intensive multimodal therapy (**Table 7**).

**Table 7:** Treatment-related adverse events in the TACE-HAIC combined with TKIs and PD-1 inhibitor group (group 1) versus the TACE combined with TKIs and PD-1 inhibitor group (group 2).

AE type n(%)	Group1 (n=79)	Group2 (n=90)	p
Weight loss	31 (39.2)	37 (41.1)	0.805
Decreased appetite	43 (54.4)	28 (31.1)	0.002
Fatigue	50 (63.3)	41 (45.6)	0.021
Abdominal pain	43 (54.4)	21 (23.3)	<0.001
Diarrhea	35 (44.3)	32 (35.6)	0.258
Nausea and vomiting	36 (45.5)	17 (18.9)	<0.001
Ascites	28 (35.4)	26 (28.9)	0.378
Rash	30 (37.9)	28 (31.1)	0.366
Hand-foot syndrome	19 (24.1)	25 (27.8)	0.589
Oral mucositis	22 (27.8)	13 (14.4)	0.034
Arthralgia	14 (17.7)	8 (8.9)	0.088
Bleeding event	20 (25.3)	4 (4.4)	<0.001
Immune-related pneumonitis	9 (11.4)	1 (1.1)	0.007

Additionally, exploratory subgroup analyses stratified by the specific TKI backbone (donafenib-based vs. lenvatinib-based) were performed to assess treatment heterogeneity; the detailed quantitative results are provided in **Supplementary Table 1**.

## Discussion

This real-world retrospective study demonstrates that combining locoregional therapy—either TACE or HAIC—with systemic treatment comprising TKIs and PD-1 inhibitors significantly improves survival in patients with unresectable HCC and PVTT, compared to TACE alone.

### Key findings and biological rationale

All combination regimens (TACE-TP, HAIC-TP, and TACE-HAIC-TP) yielded significantly longer OS and PFS than TACE monotherapy, reinforcing the value of a multimodal “local + systemic” approach in this high-risk population. The synergy likely stems from complementary mechanisms: TKIs suppress angiogenesis and modulate the immunosuppressive tumor microenvironment, while PD-1 inhibitors restore antitumor T-cell function (16–18). Locoregional therapies further enhance this effect—TACE induces immunogenic cell death through ischemic necrosis, whereas HAIC with FOLFOX delivers sustained cytotoxic exposure directly to both the primary tumor and PVTT, potentially achieving superior control of macrovascular invasion without triggering acute hypoxic stress (19, 20).

Notably, after PSM, the quadruplet TACE-HAIC-TP regimen showed no OS or PFS benefit over HAIC-TP, suggesting that early apparent gains in the unmatched cohort were confounded by baseline imbalances. We hypothesize that this attenuation stems from two interrelated factors. First, residual confounding likely persisted: clinicians may have assigned the intensive TACE-HAIC-TP regimen to patients with higher unmeasured tumor burden or more aggressive biology. Second—and more critically—the significantly higher toxicity of TACE-HAIC-TP probably compromised treatment sustainability, as frequent dose reductions or discontinuation of systemic therapy may have offset its initial intensity advantage. In contrast, the better tolerability of HAIC-TP likely supported more consistent long-term TKI and PD-1 inhibitor maintenance, resulting in comparable disease control over time.

Building on this clinical observation, emerging insights into tumor–immune crosstalk further clarify why intensified regimens may fail to translate PFS gains into overall survival benefit. The absence of a clear OS advantage with TACE-HAIC-TP suggests that durable antitumor immunity remains constrained by intrinsic and therapy-induced resistance mechanisms. Specifically, dysregulation of key oncogenic signaling pathways—such as PI3K-AKT-mTOR and JAK/STAT—can sustain an immunosuppressive microenvironment and impair T-cell function even in the presence of PD-1 blockade (21). Moreover, the acute hypoxic stress induced by TACE is known to activate the HIF-1α–VEGF–PD-L1 axis, which may further blunt antitumor immunity and counteract the effects of systemic immunotherapy (22). In contrast, HAIC-TP avoids embolization-related ischemia, potentially preserving a more favorable immune contexture for sustained T-cell activation. Together, these mechanisms may explain the observed dissociation between PFS and OS in intensified regimens and support the notion that regimen selection should prioritize immune-favorable pharmacodynamics over maximal cytoreduction alone.

The distinct toxicity profiles likely reflect the interplay between procedural stress and underlying liver dysfunction. Bleeding in HAIC-based regimens may arise from catheter-induced endothelial injury combined with oxaliplatin- and TKI-related coagulopathy. Gastrointestinal toxicity and systemic symptoms in intensified therapy (e.g., TACE-HAIC-TP) could stem from cumulative hepatic ischemia—TACE triggers acute hypoxia and inflammatory cytokine release, while sustained HAIC further burdens metabolic reserve in a cirrhotic liver. Peri-procedural hemodynamic fluctuations (e.g., contrast load, fluid shifts) may exacerbate splanchnic hypoperfusion, contributing to ascites or organ stress. These patterns underscore that adverse events are not merely dose-dependent but shaped by vascular physiology and procedural context—reinforcing the importance of rigorous patient selection, procedural standardization, and liver function preservation in minimizing complications among this high-risk population (8).

### Contextualization within current evidence

Our results are consistent with recent trials specifically evaluating locoregional plus systemic combinations. The LEAP-012 study (NCT04246177) reported a median OS of 22.0 months with lenvatinib plus pembrolizumab plus TACE, closely aligning with our TACE-TP (17.4 months) and HAIC-TP (20.5 months) outcomes (23). Similarly, the TALeTACE trial (ESMO GI 2025) demonstrated a median OS of 21.8 months with TACE plus TKI and PD-1 inhibitor, further validating the efficacy of this triple combination strategy. Although the EMERALD-1 study did not meet its primary endpoint, its median OS of 19.2 months with TACE plus durvalumab ± bevacizumab remains comparable to our findings (11). Collectively, these data reinforce the clinical value of integrating modern systemic therapy with locoregional interventions in advanced HCC, particularly in high-risk subgroups like PVTT.

Our findings also corroborate emerging Asian data supporting HAIC-based combinations (24). By directly comparing HAIC-TP and TACE-TP in the same cohort, we provide real-world evidence that both strategies are clinically valid, with choice guided by institutional capabilities and patient comorbidities rather than presumed efficacy differences.

### Clinical implications and future perspectives

For clinical practice, our data strongly argue against TACE monotherapy in HCC with PVTT. HAIC-TP emerges as a preferred first-line option for most patients, offering robust efficacy with a manageable safety profile and potential immunological advantages over embolization-based approaches. TACE-TP remains a practical alternative where HAIC is unavailable or contraindicated (e.g., in patients with coagulopathy).

Looking ahead, three priorities stand out:(i) Prospective randomized trials comparing HAIC-TP versus TACE-TP in PVTT-enriched populations to validate these hypothesis-generating findings;(ii) Development of predictive biomarkers—including PD-L1 expression, circulating tumor DNA dynamics, and radiomic features—to enable precision regimen selection;(iii) Exploration of adaptive treatment strategies that de-escalate or escalate therapy based on early response, thereby optimizing the risk–benefit ratio.Given the retrospective nature of our study, these findings should be interpreted as hypothesis-generating and warrant confirmation in prospective settings.

Importantly, real-world implementation must consider resource availability: while TACE is widely accessible, HAIC requires specialized infrastructure. Cost-effectiveness analyses will be essential for global scalability.

### Study limitations

The primary limitation of this study lies in its retrospective, observational design, which inherently precludes definitive causal inferences. Although propensity score matching mitigated measured confounding, residual unmeasured biases (e.g., granular performance status, nutritional status) may persist.Therefore, all observed associations, particularly regarding PFS benefits, should be interpreted with caution and viewed as hypothesis-generating rather than conclusive evidence of superiority.

Of note, the systemic ‘TP’ component comprised different tyrosine kinase inhibitors (lenvatinib or donafenib), introducing potential heterogeneity. To address this, we performed exploratory subgroup analyses stratified by TKI type (**Supplementary Table 1**). These analyses further support the robustness of our primary conclusion. Although limited by sample size and statistical power, no qualitative interaction was observed to suggest that the specific TKI backbone alters the treatment effect. Across both donafenib and lenvatinib backbones, the addition of TACE to HAIC-TP did not consistently confer a survival benefit and was associated with increased toxicity. This indicates that the favorable risk-benefit profile of HAIC-TP remains consistent regardless of the specific TKI employed.Additional limitations include variability in dosing, treatment modifications, and subsequent therapies, all of which may influence outcomes. Although the overall sample size was sufficient for primary comparisons, it may limit the power of subgroup analyses. External validation in multi-ethnic, prospective cohorts is warranted.While other factors such as the specific PD-1 inhibitor used may also contribute to heterogeneity, the consistency of our findings across the two distinct TKI backbones suggests that the observed treatment effects are primarily driven by the local-regional strategy rather than the specific systemic agents.

## Conclusion

In patients with unresectable HCC and PVTT, integrating locoregional therapy with TKI and ICI-based systemic treatment significantly improves survival over TACE alone. HAIC-TP demonstrated a favorable balance of efficacy and tolerability and may represent a preferred strategy in this observational cohort, while TACE-TP serves as a viable alternative. The intensified TACE-HAIC-TP regimen did not confer a clear survival benefit and carries increased toxicity, restricting its use to highly selected patients. Treatment decisions should be individualized, incorporating tumor biology, toxicity risks, and healthcare context. These real-world findings support prospective validation in randomized controlled trials.

## Data Availability

The raw data supporting the conclusions of this article will be made available by the authors, without undue reservation.
