# Supplementary material for: Combination of locoregional and systemic therapy for hepatocellular carcinoma with portal vein tumor thrombus: a real-world retrospective study
Source: Front Oncol. 2026 Mar 10;16:1776852. doi: 10.3389/fonc.2026.1776852 (PMC13008657; doi:10.3389/fonc.2026.1776852)
Supplement: Supplementary file 1 [file Table1.docx]

**Supplementary Table 1. Exploratory Subgroup Analysis of OS and PFS Stratified by Systemic Therapy Backbone (Donafenib-based vs. Lenvatinib-based).**

| Subgroup | Treatment | N | Median_OS | HR_OS_CI | P_OS | Median_PFS | HR_PFS_CI | P_PFS |
| --- | --- | --- | --- | --- | --- | --- | --- | --- |
| Donafenib-based | TACE-HAIC-TP | 43 | 19.7 | 1.00 (reference) | reference | 15.7 | 1.00 (reference) | reference |
|  | HAIC-TP | 47 | 26.1 | 0.71 (0.41-1.23) | 0.221 | 10.93 | 1.29 (0.8-2.08) | 0.304 |
|  | TACE-TP | 44 | 18.97 | 1.16 (0.71-1.92) | 0.552 | 10.63 | 1.3 (0.8-2.12) | 0.292 |
| Lenvatinib-based | TACE-HAIC-TP | 36 | 21.87 | 1.00 (reference) | reference | 13.78 | 1.00 (reference) | reference |
|  | HAIC-TP | 51 | 19.17 | 1.19 (0.67-2.12) | 0.55 | 9.73 | 1.78 (1.07-2.94) | 0.056 |
|  | TACE-TP | 46 | 16.85 | 1.42 (0.81-2.48) | 0.223 | 9.88 | 1.78 (1.06-3) | 0.061 |

*OS, overall survival; PFS, progression-free survival; HR, hazard ratio; CI, confidence interval; TACE, transcatheter arterial chemoembolization; HAIC, hepatic arterial infusion chemotherapy; TP, toripalimab plus lenvatinib/donafenib (specific TKI context).*
